# Prof. John R. Allen, M. D.

**Published:** 1855-07

**Authors:** 


### PROF. JOHN R. ALLEN, M. D.

Prof. E. R. Ford, having relinquished the practice of medicine to engage in other pursuits, tendered his rosignation as Prof. of Obstetrics and Diseases of Women and Children. He was then elected Emeritus Prof. and Lecturer on Medical Jurisprudence, which position he can fill without conflicting with his other pursuits.

To fill the vacancy then occasioned by his resignation, the Faculty have appointed the distinguished gentleman at the head of this notice.

Prof. Allen occupied for a number of years the Professorship of Materia Medica, in the Medical School at Lexington, Kentucky, with honor to himself, and the most entire satisfaction to the different classes. No member of the Faculty of that deservedly popular institution, sustained himself better in the department to which he was assigned than did Prof. Allen, and no one added more to the popularity and prosperity of the school than was rendered by his talents and his efforts.

During his connection with the above Institution, he was selected and assigned by the State authorities to the charge of the Lunatic Asylum of that State, which position he held for many years, during which time, that Institution was in the most prosperous state and condition. Meanwhile the cholera madeits appearance among the unfortunate inmates, and although we could conceive of nothing more apalling than the presence of such a disease among the insane, yet he labored with them constantly, by night and by day, and until the disease subsided, with untiring assiduity and a humane devotion to their best interests.

Subsequently he was elected Prof. of Obstetrics in the Medical Department of the Missouri University at St. Louis, and having secured valuable interests in this city and county, with his family he removed to this place.

Prof. Allen has the reputation of an accomplished teacher and lecturer, beside being a profoundly scientific man. As he has selected this city as his future home, where he will engage in the practice of his profession, this Institution will find in him an important acquisition, the profession a valuable addition, and society a valuable member.

He has brought with him very many valuable and most interesting appliances for the full illustration of his branch, so that we can promise the class of the coming session that the next course, through the arrangements in progress in this Institution, will prove to be the most interesting and profitable of any previous session, and will fully answer the fondest wishes and highest expectations of those who will attend for the laudable purpose and object of scientific improvement.

